# Editorial: Multicellularity: Views from cellular signaling and mechanics

**DOI:** 10.3389/fcell.2023.1172921

**Published:** 2023-03-13

**Authors:** Hiroaki Hirata, Naotaka Nakazawa, Tsuyoshi Hirashima, Andrea Ravasio

**Affiliations:** ^1^ Department of Applied Bioscience, Kanazawa Institute of Technology, Hakusan, Japan; ^2^ Department of Energy and Materials, Faculty of Science and Engineering, Kindai University, Higashiosaka, Japan; ^3^ Mechanobiology Institute, National University of Singapore, Singapore, Singapore; ^4^ Institute for Biological and Medical Engineering, Schools of Engineering, Medicine and Biological Sciences, Pontificia Universidad Católica de Chile, Santiago, Chile

**Keywords:** cell-cell signaling, cell-cell communication, multicellular biomechanics, multicellular mechanobiology, morphogenesis, collective cell dynamics

Appearance of multicellularity is a milestone for the evolution of life on our planet (Rose and Hammerschmidt, 2021). Multicellular organisms display emergent properties that cannot be predicted from simple cellular aggregation. Such properties stem from regulated mechanical interaction and dynamic communication between cells and between cells and their environment. Indeed, recent works have demonstrated that integration of mechano-chemical signaling is essential to specify cellular activity and maintain tissue homeostasis during key biological processes such as tissue morphogenesis, collective cell migration, cell differentiation and cell proliferation (Hannezo and Heisenberg, 2019). On the other hand, dysregulation of cell-cell communication and organization can cause fatal system failures and diseases, e.g., inappropriate execution of developmental processes and cancer transformation and progression (Northcott et al., 2018; Hannezo and Heisenberg, 2019).

To mechanistically understand how forms and functions of multicellular bodies arise, we need to know 1) mechanical and biological properties of individual constituent cells, and 2) how these properties are integrated into the whole system. Another to-be-emphasized aspect in multicellular systems is that mechanics and dynamics of individual cells are largely modulated by cues of the cell’s environment, and local chemical and mechanical environments in the systems are, in turn, modified by cellular activities. Thus, for the understanding of multicellular systems, it is also requisite to unveil the mechanisms for reciprocal regulations between cells and environments. This Research Topic, with a series of nine articles from both experimental and theoretical approaches, highlights how mechanics-signaling integration in cells and their environments contributes to structures and functions of multicellular systems.

Development and maintenance of multicellular systems rely on adhesion of cells to their surrounding extracellular matrices and neighboring cells through tightly regulated interfacial protein complexes. Notably, the cell adhesion machineries suffer mechanical loads originated by biological activities and mechanics in both intracellular and extracellular spaces. Lim et al. comprehensively review molecular bases of “mechanotransduction” (i.e., transduction of mechanical stimuli into biochemical signals and vice versa) at cell-extracellular matrix (ECM) and cell-cell adhesions, especially focusing on their impacts on the regulation of stem cell functions in their niche. Ravasio et al. propose a novel concept of “mechanoautophagy” by discussing potential synergies between mechanotransduction at cell adhesions complexes and autophagy, a regulated catabolic process used by cells to respond to a variety of cellular stresses, including those of mechanical nature.

Biswas et al. and Hirashima provide state-of-the-art reviews on integration of mechanical and biochemical signals as principles of multicellular tissue morphogenesis. Focusing on the process of egg formation in the ovarian follicle (folliculogenesis), Biswas et al. discuss how a range of crosstalks between local mechanical cues (e.g., ECM mechanics, luminal hydrostatic pressure, and cell-generated forces) and intracellular signaling (e.g., Hippo and Akt pathways) contribute to individual stages of follicle development. Furthermore, they summarize emerging experimental technologies employed for mechanobiological analyses in ovarian biology. On the other hand, Hirashima highlights importance of mechano-chemical feedback mechanisms for size regulation of epithelial tissues. Specifically, he discusses how the homeostasis of the epithelial tissue volume and the epithelial tube diameter is achieved by the control of cell division and cell rearrangement through the mutual feedback between cell-cell force communication and intracellular signal transduction.

The effect of ECM mechanics on the interaction between cell populations is revealed by Aparicio-Yuste et al. Epithelial cells infected with bacteria are known to be extruded from epithelial tissues through a mechanism called “cell competition”, which involves competition between infected and surrounding uninfected cells. Using a combination of experimental and computational approaches, the authors show that a stiffer ECM promotes cell competition-mediated extrusion of infected cells by preferentially stimulating protrusive activities of uninfected cells.

Extensive efforts have long been made, from a wide range of experimental and theoretical approaches, to understand how body axes and patterns are developed in early embryos. In this Research Topic, three research groups develop new computational models to explore mechanical factors that contribute to body axis/pattern formation during embryonic development. Fujiwara et al. developed a simulation framework for a multicellular sphere based on vertex model, which is well-suitable for computational analysis of the body axis formation process. By incorporating parameters corresponding to cell-cell interfacial tension, without considering local control of cell division rate/orientation, they succeed to reproduce morphological and gene expression patterns observed during body axis elongation. Koyama et al. theoretically find that mismatches in viscous friction and cell area elasticity between two adjacent cell populations can lead to morphological changes similar to those observed in developing mouse embryos. Consistent with this finding, they experimentally confirm through AFM measurement that two contacting cell clusters during notochord elongation in mouse embryos exhibit varying cellular stiffness. Finally, Montenegro-Rojas et al. used early embryonic development of annual killifish as a model to computationally investigate the minimal mechanical requirements for *de-novo* formation of a cell aggregate. In this work, the authors developed a novel *in silico* framework that, combined with a bottom-up approach, allowed them to scan for those mechanical parameters (e.g., cell migration, modulation of cell-cell adhesion and of contact inhibition of locomotion and taxis mechanisms) necessary to maintain embryonic cells in dispersion on the surface of the yolk and thereafter aggregate at the embryo´s pole to initiate gastrulation.

The mechanism of multicellular pattern formation in embryos is addressed also using a gene expression analysis approach. Using undifferentiated cells and their nuclei dissociated from the early spider embryo, Akiyama-Oda et al. conducted single-cell and single-nucleus RNA sequencing and successfully reconstructed cell populations that correspond to the ectoderm, mesoderm and endoderm, as well as the cell patterning along the anterior-posterior axis in the ectoderm of the embryo. The transcriptome data resources provided in this study would be useful for further studies that aim at comprehensive identification of new patter-generating genes whose expression is spatially controlled.

The insights provided by the articles in this Research Topic contribute to a better understanding of mechano-chemical principles for emergence of multicellular functions and they highlight the importance of multidisciplinary investigation to unravel the intrinsic complexity of such systems. Indeed, while mono-disciplinary investigations explore the depth of the inner workings of living beings, combination of perspectives and technologies borrowed from different disciplines has provided the material and intellectual tools to navigate complexity found in multicellular organisms (Thomas et al., 2004; Grenci et al., 2019; Kim et al., 2020). On the same line, it is crucial to integrate approaches based on *in vivo* investigation, where physiological and pathological scenarios present themselves in their intricate completeness, with *in vitro* reductionist approaches where hypothesis testing can be based on simple assumptions and leverage from accurate control of experimental conditions as well as unparalleled precision and resolution. Finally, physical or statistical-based *in silico* approaches can be used to generate computational models and the same models can be further used to design informed investigations. Similarly, it seems natural to suggest that, due to the intrinsic complexity of the system, the plethora of approaches required, and the variety of results obtained, it will be increasingly necessary to adopt advanced statistical and computational methods such as Machine Learning and Artificial Intelligence to further advance the field. In conclusion, it is our shared opinion that the works presented here provide a substantial contribution to advancing our knowledge on how multicellular systems work in both physiological and pathological situations. Importantly, this may bring clues of novel strategies for engineering and medical application of multicellular systems, such as designable and efficient tissue engineering methods for regenerative medicine.
